# Force Myography-Based Human Robot Interactions via Deep Domain Adaptation and Generalization

**DOI:** 10.3390/s22010211

**Published:** 2021-12-29

**Authors:** Umme Zakia, Carlo Menon

**Affiliations:** 1 Menrva Research Group, Schools of Mechatronic Systems Engineering and Engineering Science, Simon Fraser University, Metro Vancouver, BC V5A 1S6, Canada; uzakia@sfu.ca; 2 Biomedical and Mobile Health Technology Laboratory, ETH Zurich, Lengghalde 5, 8008 Zurich, Switzerland

**Keywords:** force myography technique, applied force estimation in dynamic motion, transfer learning, pretrained model, domain adaptation, domain generalization

## Abstract

Estimating applied force using force myography (FMG) technique can be effective in human-robot interactions (HRI) using data-driven models. A model predicts well when adequate training and evaluation are observed in same session, which is sometimes time consuming and impractical. In real scenarios, a pretrained transfer learning model predicting forces quickly once fine-tuned to target distribution would be a favorable choice and hence needs to be examined. Therefore, in this study a unified supervised FMG-based deep transfer learner (SFMG-DTL) model using CNN architecture was pretrained with multiple sessions FMG source data (D_s_, T_s_) and evaluated in estimating forces in separate target domains (D_t_, T_t_) via supervised domain adaptation (SDA) and supervised domain generalization (SDG). For SDA, case (i) intra-subject evaluation (D_s_ ≠ D_t-SDA_, T_s_ ≈ T_t-SDA_) was examined, while for SDG, case (ii) cross-subject evaluation (D_s_ ≠ D_t-SDG_, T_s_ ≠ T_t-SDG_) was examined. Fine tuning with few “target training data” calibrated the model effectively towards target adaptation. The proposed SFMG-DTL model performed better with higher estimation accuracies and lower errors (R^2^ ≥ 88%, NRMSE ≤ 0.6) in both cases. These results reveal that interactive force estimations via transfer learning will improve daily HRI experiences where “target training data” is limited, or faster adaptation is required.

## 1. Introduction

Force myography is a contemporary, non-invasive, wearable technology like the traditional surface electromyography (sEMG) and can read muscle contractions without requiring skin preparations or precautions. This technology is based on force sensing resistors (FSRs) that detect resistance changes when pressure is applied to them. An FMG band donned around a limb on the upper or lower extremities can be used to detect underlying muscle contractions during activities, and these signals can be interpreted using machine learning (ML) techniques [1]. Although sEMG technology has been around for several decades, the measured electrical activities of underlying muscles during movements of limbs are faint, requiring substantial and costly signal processing units and skin preparation for electrode placements [2]. In contrast, FMG technique is cost effective, repeatable, electrically robust, and requires minimal signal processing and optional feature engineering [3]. In addition, FMG technique was found effective, like sEMG, in several research studies [4,5,6,7] as an emerging technology and has been studied in similar applications of gesture recognition, prosthetic control, activities of daily life, rehabilitation, and human machine interactions (HMI) [8,9,10,11,12,13]. However, there are very few studies on FMG-based deep transfer learning (DL) techniques in human robot interactions (HRI). In a recent study, transfer learning for hand gesture classification using convolutional neural network (CNN) via FMG signals was investigated [14]. Authors in [15] showed improved gesture recognition accuracy via FMG-based transfer learning by incorporating multiple source domains from other persons. Only in one study on FMG-based pHRI, researchers implemented an FMG-based recurrent neural network (RNN) model to classify whether a hand movement pattern in a collaborative task with an industrial robot was user intended or random [16].

As an established technology, sEMG has been well studied in implementing deep transfer learning in HMI, HRI, and other applications. Like FMG, this signal is affected by electrode placement on the limb, electrode shift, intensity, and variability in muscle contraction during intra-/inter-session evaluation, limb motion, or postures during interactions [17]. Despite the transient nature of the signal, studies conducted on model generalization and domain adaptation showed impressive results [18,19]. Researchers trained a model with inductive and supervised transductive transfer learning for gesture classification using a random forest (RF) algorithm; the model was fine-tuned with short calibration data from an unseen participant and evaluated successfully with a humanoid Pepper robot [20]. High-density sEMG (HD-sEMG) signals were used for a deep domain adaptation framework and were found to improve inter-session gesture detection for unlabeled test data or fine-tuning of labelled calibration data [21]. Electrode shifts and day-to-day variability through adaptive transfer learning were investigated thoroughly [22,23]. Moreover, periodic recalibration with a small amount of training data was found effective in multiple days usage for prosthetic control by applying transfer learning [24]. In [25], the domain shift between data in each training trial (source domain) was evaluated for cross-subject elbow EMG-torque models and calibration data acquired from a new subject (target domain) using feature correlation. In [26], authors proposed supervised covariate shift adaption method using a small calibration set only. Furthermore, a recent study showed that aggregating source distributions from multiple users with deep transfer learning in gesture recognition enhanced the model’s performance [27]. Among the sEMG-based pHRI studies in the literature, few approached applied force and torque in dynamic motion because of complexities with regressions when calibrations were required. Since the FMG signal has similar characteristics, and there is a gap in the literature using transfer learning for the pHRI regression problem, this study focuses on force estimation using multiple session data sets via transfer learning.

In most industrial human-robot collaborative activities, applied hand forces are required to carry on certain tasks. Traditional force/torque (FT) sensors can read the applied force precisely although these are bulky, require special signal processing units, and are uncomfortable as a worn device. Estimating isometric or dynamic hand force/torque using FMG signals via FSRs were found favorable to FT sensors [28,29,30]. Recently, measuring force via FMG bio signals during physical human robot interactions (pHRI) between human participants and a linear robot was found effective for intra-session evaluation using traditional ML algorithms [31]. However, intra-session FMG-based pHRI required collecting adequate labelled training data, which was biased and impractical in real scenarios. In addition, each session data was affected by transient, instantaneous signals, sensor position shift, physiological changes, limb motions, and postures each time an FMG band was donned. Such domain shifts and lack of adequate data severely limited inter-session or inter-participant performance evaluations. In a recent study, inter-participant domain generalization via traditional support vector regressor (SVR) was investigated [32] although this study did not investigate deep transfer learning or intra-session evaluations when a participant interacted with a robot on regular basis.

Therefore, this study conducted a major investigation for feasibility of FMG-based HMI and HRI applications via deep transfer learning where interactions were expected to occur on regular basis. Transductive transfer learning (few target data available/seen) via supervised domain adaptation (SDA) for inter-session evaluation and inductive transfer learning (target data not available/unseen) via supervised domain generalization (SDG) for inter-participant evaluation was investigated to overcome limitations of intra-session evaluation [33,34,35]. Domain adaptation reuses part of a model pretrained with large pools of source domains to predict different but related target domain where both domains have same feature spaces with different distributions. On the other hand, domain generalization uses a pretrained model with source domains and attempts to predict unseen target data. It is particularly beneficial to mitigate gaps between different domains where knowledge about the target domain is absent [36,37]. These methods have been successfully applied in image processing, but there are very few studies in bio-signal-based pHRI because of transient and dynamic nature of bio feedback and hence needs to be investigated. In a repetitive FMG-based pHRI application between a participant and a robot, previous intra-sessions data could contribute building a large dataset. Due to the transient signal, sensors shift, and dynamic interactive environment, each session’s data were unique even when the task (applied force in certain motion) was the same. Therefore, the focus of this study was to investigate whether these multiple-source data could improve the user experience in daily interactions utilizing domain adaptation by pretraining a model and fine-tune via transfer learning. We further investigated the impact of domain generalization for a different pHRI task between the robot and several other participants (applied interacting force in another motion) using the same pretrained model. Such cross-subject evaluation became more challenging due to signal variability between the target distribution and the multiple intra-sessions source distributions. Fine-tuning the pretrained model via transfer learning could leverage the gap between the source and target domain. For both SDA and SDG, few calibration data (target training data) was used for fine-tuning the model to adapt instantaneous state of the signal captured during the dynamic interactions.

**Figure 1:**
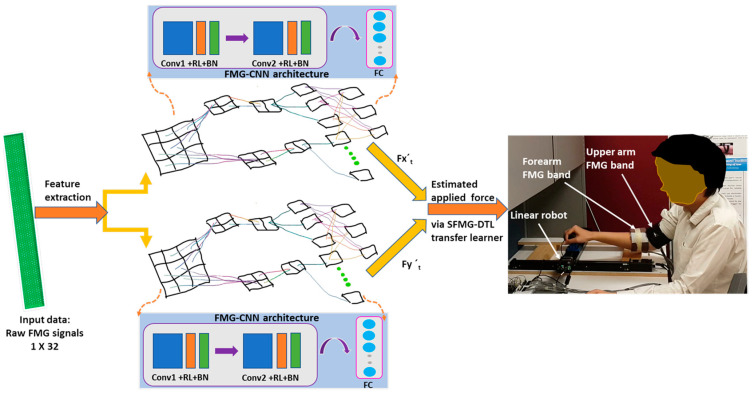
The proposed SFMG-DTL transfer learning model for estimating applied force during pHRI on a planar workspace with a linear robot.

An FMG-based convolutional neural network (FMG-CNN) architecture was proposed to investigate pHRI between several human participants and a linear robot/stage via domain adaptation and generalization. This architecture was used as a nonlinear regression model to map applied forces from instantaneous FMG signals during interactions, as shown in Figure 1. For transfer learning, multiple source distributions were used to pretrain a unified supervised FMG-based deep transfer learner (SFMG-DTL) model during the training phase. These multiple sources of FMG distributions (source distribution: D_s_) were collected in several sessions during regular pHRI activities between one human participant and the linear robot while the participant applied hand forces in a certain dynamic SQ-1 motion (source task: T_s_). The SFMG-DTL model was assessed on separate cases during the evaluation phase on target domain 1 for supervised domain adaptation (case i: SDA) and on target domain 2 for supervised domain generalization (case ii: SDG). In case i, inter-session target domain 1 (D_t-SDA_) was evaluated where the same participant (intra-subject) interacted with the linear robot in SQ-1 motion (T_t-SDA_). While in case ii, inter-participant target domain 2 (D_t-SDG_) was assessed separately for five (5) other participants (cross-subject) interacting with the linear robot in SQ-2 motions (T_t-SDG_). In the beginning of evaluation for both cases, a few calibration data (target training data) were collected to fine-tune the pretrained model in recognizing target distribution. Intra-session evaluations on target domains (target training and target test data) were conducted using FMG-CNN architecture for comparing performances of SDA and SDG cases. Several machine learning algorithms, such as support vector regression (SVR) and multi-dimensional support vector regression (MSVR), were also used for performance comparison in domain adaptation.

Major contributions of this study were:

- Investigating feasibility of deep transfer learning technique in repetitive FMG-based pHRI applications utilizing inter-session FMG data for the first time;
- Proposing a unified transfer learner for both supervised domain adaptation and domain generalization;
- Leveraging periodical calibration as needed with less data than normally required; and
- Proposing a nonlinear FMG-CNN regression architecture for mapping applied force from FMG signals without requiring biomechanical modelling of the human arm.

The rest of this article is organized as follows: Section 2 describes the materials and methods, where methodology, experimental setup, and protocol used are explained. Results are discussed in Section 3. Performance evaluations of the proposed framework is discussed in Section 4, while Section 5 concludes this article.

## 2. Materials and Methods

### 2.1. Problem Statement

#### 2.1.1. Source and Target Domain

In this study, multiple source domains, *D_si_* = {*i* = 1, 2, 3}, were used for pretraining a deep transfer learning model. Source domain *D_si_* = {*χ_sj_, Y_sj_*} had data matrix *χ_sj_* ∈ *R^Nsj^* *× S^C^* such that *i* ∈ {1, 2, 3}, *j* ∈ {1, 2, *...*, *N_S_*}, *S^C^ =* {*c*_1_*, ..., c*_32_} (*c*: 32 FMG channels, *S^C^* = dimensionality of feature vectors, and *N_S_*: number of samples), and labels *Y_sj_* = {*F_sjx_, F_sjy_, f* (·)} [*f* (·) was a predictive function, and *F_sjx_*, *F_sjy_* were label space of applied forces in X and Y dimensions such that *f:* *χ_sj_ → F_sj-x_* and *f :* *χ_sj_ → F_sjy_*]. All distributions were homogenous and balanced. Target domain *D_t_ =* {*χ_t_*} had data matrix *χ_t_* ∈ *R^Nt^* *× S^C^* [*S^C^*: dimensionality of feature vectors, and *N_t_*: number of samples in target domain]. Calibration data, C_d_ ∈ {X_c_, Y_c_} [*Y_c_ =* {*F_cx_, F_cy_, f* (·)}], a small subset of *D_t_*, was used as target training data. A transfer learner pretrained with *D_si_* and fine-tuned with *C_d_* predicted force label spaces, *Y_t_ =* {*F_tx_, F_ty_, f* (*·*)}, from target test distribution: {χ_t_^*^} ∈ *D_t_*. In case of domain adaptation, source and target domains were different, but source and target tasks of applied force estimations in SQ-1 motion were same (*D_s_ ≠ D_t_, T_s_ = T_t_*) {*T_s_, T_t_*: applied interactive forces in SQ-1 motion}). While in domain generalization, both source and target domains and tasks were different (*D_s_ ≠ D_t_,* and *T_s_ ≠ T_t_*, where *T_s_*: applied forces in SQ-1 motion and *T_t_*: applied force in ‘SQ-2′ motion). Acronyms used in this article are listed in Table 1.

#### 2.1.2. Applied Interaction Force Estimation

At an instant of time, t, instantaneous raw input target test signals *S^C^* arriving at the model (with a *δ* of *µ* parameter set) with a probability *P_t_* (*S_t_^C^*) mapped estimated applied force *F_xt_^’^* and *F_yt_^’^* (forces in X and Y dimensions) in a dynamic motion such that:

(1) $$f_{x}\left(\cdot \right)=F_{xt}^{\prime }=\delta ,\left(s_{t}^{C},\mu_{1}\right)$$

(2) $$\;\;f_{y}\left(\cdot \right)=F_{yt}^{\prime }=\delta ,\left(s_{t}^{C},\mu_{2}\right)\;$$

To find best parameter space *µ*, loss function was computed:

(3) $$\mu_{1}=\;L\left(F_{xt}^{\prime }-F_{xt}\;\right)=\arg\underset{µ1}{\min}\sum_{k=1}^{t}{\left(\;F_{xk}-F_{xk}^{\prime }\right)}^{2}$$

(4) $$\mu_{2}=\;L\left(F_{yt}^{\prime }-F_{yt}\;\right)=\arg\underset{µ2}{\min}\sum_{k=1}^{t}{\left(\;F_{yk}-\;F_{yk}^{\prime }\;\right)}^{2}\;$$

Mean square error (*MSE*) was used to calculate average squared difference between estimated and real value. *MSE* for a single observation was:

(5) $$MSE_{x}=\sum_{k=1}^{R}\frac{{\left(\;F_{xk}-F_{xk}^{\prime }\right)}^{2}}{R}$$

(6) $$MSE_{y}=\sum_{k=1}^{R}\frac{{\left(\;F_{yk}-F_{yk}^{\prime }\right)}^{2}}{R}\;$$

where *R* was the number of responses; *F_xk_*, *F_yk_* were the target output; and *F_xk_’*, *F_yk_’* were the network’s prediction for response *k*. 

### 2.2. Experimental Setup

**Figure 2:**
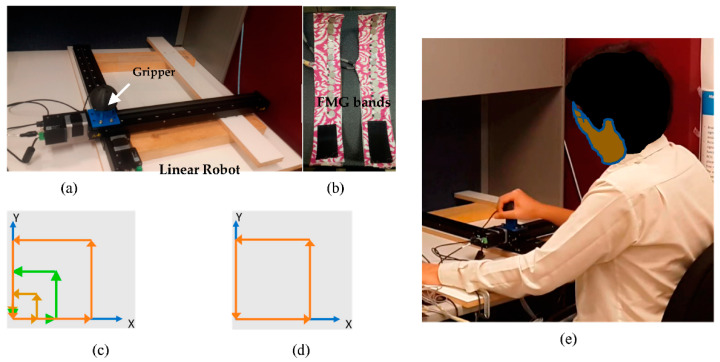
Setup used for data collection and evaluation of SFMG-DTL: (**a**) linear robot with gripper and end-effector on top, (**b**) two FMG bands, (**c**) interaction force in square motion SQ-1 with variable sizes in domain adaptation, (**d**) interaction force in square motion SQ-2 in domain generalization, and (**e**) participant P_1_ interacting with the robot by applying force in dynamic SQ-1 motion.

FMG-based pHRI was investigated where a human participant collaborated with a linear robot/biaxial stage, as shown in Figure 2. Interactions occurred by applying force at the end-effector of the robot. Two FMG bands (32 feature space) using FSRs (TPE 502C, Tangio Printed Electronics, North Vancouver, BC, Canada) were used to read muscle contractions during interactions using data acquisition systems (NI DAQs 6259, 6341, National Instruments, Austin, TX, USA). These bands were wrapped around the forearm and upper arm muscle belly. A customized linear robot or a cartesian planar robot had two perpendicular linear stages (X-LSQ450B, Zaber Technologies, Vancouver, BC, Canada) in the X and Y dimensions on the planar workspace with a customized gripper on top as the end-effector. The true label of applied force was recorded with a 6-axis FT sensor (Mini45, ATI Industrial Automation, Apex, NC, USA) that was mounted inside the gripper. Compliant collaboration was implemented via admittance control where the applied force was converted proportionally to the motor displacements of the linear stages. Therefore, the gripper would slide along the workspace, following the same trajectory of the human-applied force in dynamic motion and direction. The linear robot was fixed firmly on a table for interactions. An HP Zbook laptop (Intel core i7, 16GB RAM) was used for data collection via Labview interface and for model evaluations via Matlab scripts.

### 2.3. Proposed FMG-CNN Architecture

**Figure 3:**
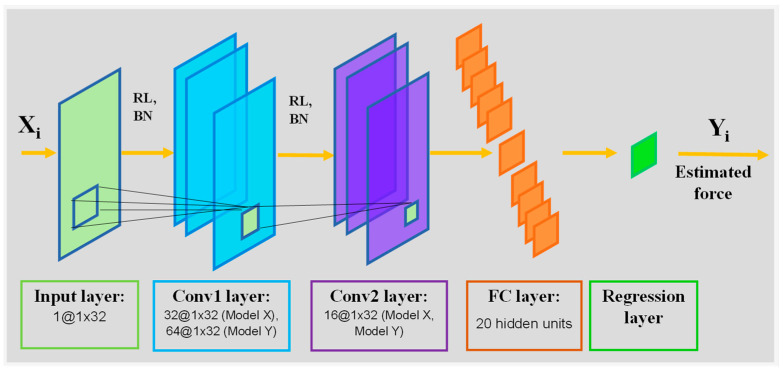
Proposed FMG-CNN architecture (Model X, Y).

Figure 3 shows the proposed FMG-CNN architecture used in this study. Raw FMG signals were used for training and evaluating SDA and SDG. Two separate models had an input layer of input size 1 × 32 with “zerocenter” normalization, followed by Model X and Model Y, used for estimating forces in X and Y dimensions. Both model conv1 and conv2 convolutional blocks. Raw data was preprocessed using minmax scaling before passing to the input layer. In each convolution block, the conv layer was followed by a Relu and a batch normalization layer. For Model X, 32 filters were used in the conv1 block, while 64 filters were used for Model Y. The conv2 layer had 16 filters in both models. A fully connected layer with 20 connections followed the conv layers, and finally, a regression layer was used to map the instant force. Batch normalization helped to alleviate the internal covariance shifting present during training, as changes happened in input distributions of layers due to parameter changes in previous layers. Filters sized 3 × 3 with a stride of 1 and a padding of 1 was used. During evaluation, fine-tuning occurred in the final fully connected layer. For both pretraining and fine-tuning, stochastic gradient descent (SGD) was implemented as the optimizer. A learning rate (LR) of 1E-04 and maximum epoch (E) of 40 were used in pretraining, while LR = 1E-05 with E = 60 was used during evaluation. MSE loss was used for validation of the training process.

**Figure 4:**
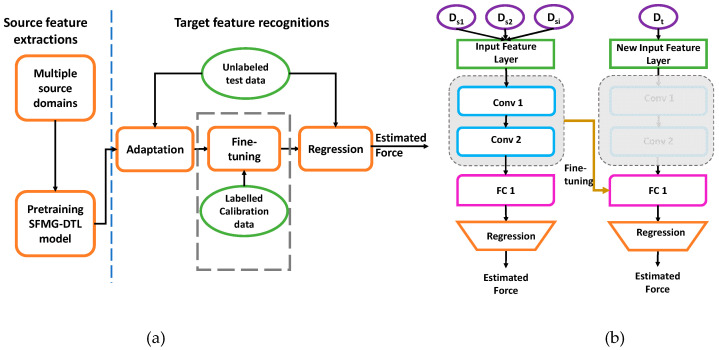
FMG-based transfer learning: (**a**) estimating applied interactive forces via SDA and SDG and (**b**) fine-tuning process of the pretrained SFMG-DTL model.

For transfer learning, a unified framework for SDA and SDG based on the FMG-CNN architecture was proposed, as shown in Figure 4. In this framework, the model learned discriminative features of the multiple source domains during pretraining. While fine tuning, the last three layers of the saved model helped in adapting to converge quickly in recognizing target distribution.

### 2.4. Protocol

A total of 6 participants (P_1_, …, P_6_) volunteered in this study. All participants were healthy, right-handed, and their average age was 33 ± 8 years. Informed consents were obtained from all subjects involved in the study, as approved by Office of Research Ethics, Simon Fraser University, British Columbia, Canada.

**Figure 5:**
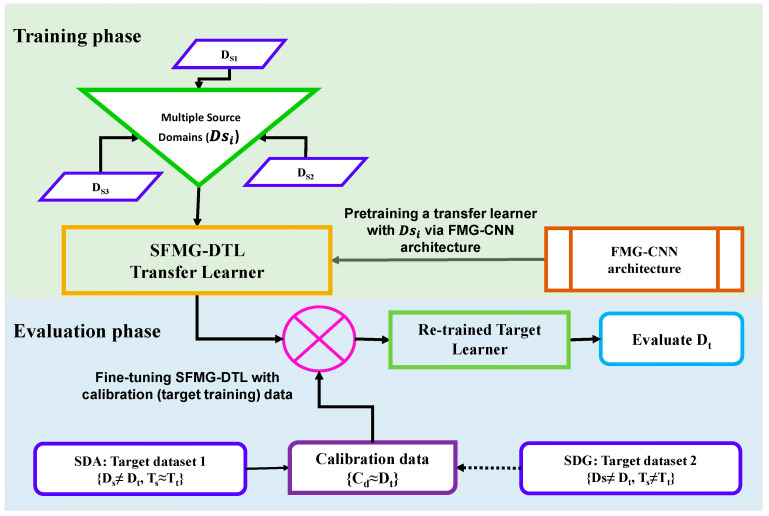
SFMG-DTL: unified transfer learning framework for SDA and SDG.

Figure 5 shows the training and evaluation phases followed in this study to investigate the proposed SFMG-DTL transfer learning model. Both source and target distributions and model hyper parameters used are summarized in Table 2. During the training phase, source distributions were collected and used for pretraining the model, while in the evaluation phase, separate target domains for SDA and SDG were collected and evaluated separately, as discussed below.

#### 2.4.1. Training Phase

##### *Multiple-Source* *Data Collection*

Multiple training data collection sessions were conducted in three (3) different sessions during interactions between participant P_1_ and the linear robot. The collaborative task was conducted by applying hand force in a dynamic square motion SQ-1 of varying sizes on the planar surface, as shown in Figure 2. Participant P_1_ sat in front of the linear robot/biaxial stage comfortably on a chair locked in position.

Two FMG bands were donned on the forearm and upper arm on the participant’s dominant right hand (Figure 1 and Figure 2e). A total 14 cycles of data were collected during these sessions, where 600 × 32 samples of data were collected in a cycle. In each cycle, participant grasped the gripper and applied interactive force in a dynamic square motion, defined as the source task (T_SDA_ = applied force in SQ-1 motion). Applying forces in a non-uniform anti-clockwise square motion with gradually increasing displacement area on the planar surface (Figure 2c) were repeated continuously to complete one cycle.

##### *Pretraining Deep* *Learning Model*

For domain adaptation and generalization, the proposed FMG-CNN architecture was used for pretraining the unified SFMG-DTL transfer learner model. The model was trained to predict applied forces in X and Y dimensions simultaneously from a distribution. Two separate models (Model X, Model Y) were generated for estimating forces in X and Y dimensions and saved as .mat file for use in evaluation sessions.

#### 2.4.2. Evaluation Phase

##### *Case i: Evaluating Intra-Subject/Inter-Session* *Target Domain* (*D_t-SDA_, T_t-SDA_*) *via* *Domain Adaptation* (*D_s_* *≠ D_t_, T_s_ ≈ T_t_*)

Inter-session evaluation was investigated to see if multiple session data from a repetitive user (intra-subject/participant) could be useful in practical applications. In this target task, participant P_1_ interacted with the linear robot in similar motion speed and pattern SQ-1 following same source data collection protocol. For domain adaptation, first, a few calibration data were collected as target training data (1200 × 32 samples) for fine-tuning and formed target dataset 1. The transfer learner was thus retrained to adapt a new target domain. It was then evaluated on 400 × 32 samples of target test data.

##### *Case ii:* *Evaluating Cross-Subject/Inter-Participant Target Domain* (*D_t-SDG_, T_t-SDG_*) *via* *Domain Generalization* (*D_s_* *≠ D_t_, T_s_* *≠ T_t_*)

For domain generalization, five participants (P_2_:P_6_) contributed to evaluate the pretrained SFMG-DTL model. Target distributions were collected from each participant during a collaborative task that allowed interaction with the robot applying force in a uniform square motion (T_SDG_ = applied force in SQ-2 motion), as shown in Figure 2d. For each participant, a total 4 cycles of target data (400 × 32 samples/cycle) were collected with similar source data collection protocol, and it was termed as target dataset 2. Leaving one out cross-validation (LOOCV) was implemented where 3 cycles were used as target training data for fine-tuning the SFMG-DTL model, and 1 cycle was used as target test data.

### 2.5. Performance Matrices

#### 2.5.1. Statistical Tools and Tests

Performance of the SFMG-DTL model in estimating force in the dynamic motion was evaluated using the coefficient of determination (R^2^) and normalized root mean square error (NRMSE).

Coefficient of determination (R^2^) was obtained by:

(7) $$R^{2}=\frac{Explained\;variation}{Total\;variance}$$

It was used to determine the correlations or dependencies of the dependent variable on the independent variable. R^2^ or goodness of fit values varied between 0 and 1.

NRMSE determined the fraction of RMSE (squared root of differences between predicted and real value) to the observed range of the measured data:

(8) $$NRMSE=\frac{\sqrt{\frac{1}{n}\Sigma_{i}{\left(Y_{e}-Y_{i}\right)}^{2}}}{mean\left(Y\right)}$$

where Y was the measured data, n was number of samples, and Y_e_ was the prediction made by the regression model.

A *t*-test was performed to evaluate effectiveness of domain generalization. It was a statistical test to compare the means of two samples to determine the significance in change [38]. It helped to determine whether performance improvement using transfer learning with the SFMG-DTL model was statistically significant.

#### 2.5.2. ML and DL Algorithms

For performance evaluation of SFMG-DTL model, intra-session evaluations were conducted on the two target domains using baseline FMG-CNN architecture. For intra-session evaluation, a baseline SDA and a baseline SDG model were trained with target training data and evaluated on the same target test data (as mentioned in Section 2.4.2 and Table 2). Intra-session evaluation used SGD optimizer and hyper parameters (LR = 1E-4, E = 60) for comparable performances. A traditional machine learning algorithm, such as support vector regression (SVR) and its variation multi-dimensional support vector regression (MSVR), was used for performance evaluation of SDA only. These algorithms also used the same target training and test data for comparison with SFMG-DTL. The popular SVR model (n_u_-SVR with hyper parameters: Cost (c) = 20, Gamma (g) = 1, Epsilon (ε) = 1) could predict continuous ordered variables either in linear or non-linear way. MSVR (c = 0.01:0.5:0.09, g = 0.8:0.2:1.5, ε = 0.08) was capable of estimating force in one direction while considering forces acting in other dimensions. For MSVR, instead of using separate model estimating force in each dimension, a single model was trained to predict forces. This model was investigated to determine if higher accuracies could be achieved while reducing computation resources and time. For both SVR and MSVR, best values for cost (c) and gamma (g) were obtained by grid searches. Separate models were generated to predict forces in the X and Y dimensions for SVR, intra-session, and SFMG-DTL, while only one MSVR model was trained for predicting forces in both dimensions. All models utilized radial basis function (RBF) kernel.

## 3. Results

**Figure 6:**
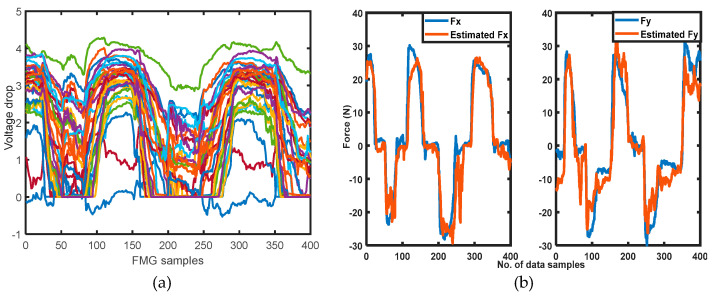
Target dataset 1 used in SDA: (**a**) target test FMG data; (**b**) true forces and estimated forces in X and Y dimensions estimated by the retrained SFMG-DTL learner.

For transfer learning in SDA and SDG, the SFMG-DTL pretrained model was evaluated with two separated target domains (in both cases, calibration data/target training data (1200 × 32 samples) and target test data (400 × 32 samples) were of same amount). Figure 6 shows plots of target domain 1: FMG test distributions and the model’s performance of force estimations in X and Y dimensions during SDA.

### 3.1. Supervised Domain Adaptation

**Figure 7:**
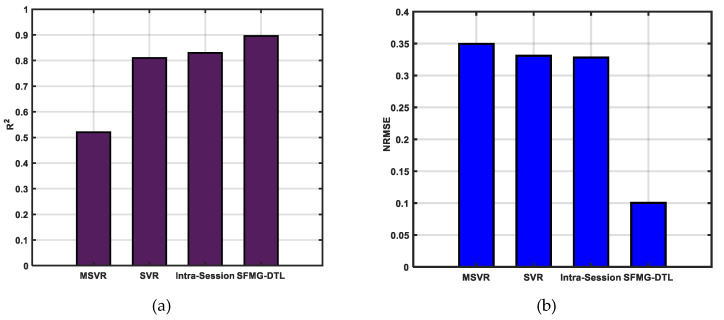
Performances of ML and DL models in case i: on target dataset 1 (supervised domain adaptation): (**a**) estimation accuracies (R^2^) and (**b**) error in estimation (NRMSE). Averaged values (Model X and Model Y) are reported for SVR, intra-session, and SFMG-DTL.

Supervised domain adaptation was investigated for inter-session FMG data for repetitive pHRI application with participant P_1_. The results obtained for R^2^ and NRMSE with the SFMG-DTL model along with other models are reported in Figure 7. The proposed deep transfer learner (MSE loss ≈ 5.8) outperformed in estimating force in the selected motion SQ-1 in terms of higher accuracies (R^2^ ≈ 89%) and lower error (NRMSE ≈ 0.10) than other algorithms, including intra-session baseline SDA’(FMG-CNN model with target training data and target test data only). Among these models, MSVR performed poorly (R^2^ ≈ 52%) despite using a single model to predict force in both X and Y dimensions. Both baseline SDA and SVR showed similar results in predicting force (R^2^ ≥ 81%). Reported values were averaged for Model X and Model Y in estimation accuracies and losses.

### 3.2. Supervised Domain Generalization

**Figure 8:**
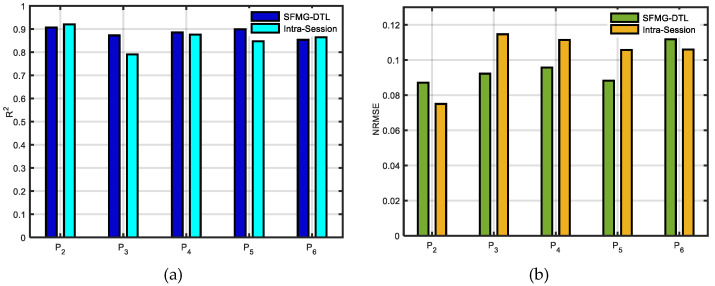
Performances of ML and DL models in case ii: on target dataset 2 (supervised domain generalization): (**a**) estimation accuracies (R^2^) and (**b**) error in estimation (NRMSE). Averaged values (Model X and Model Y) are reported for both intra-session and SFMG-DTL.

Supervised domain generalization was evaluated for inductive transfer learning where the target distributions were unseen to the pretrained model. An inter-participant/cross-subject test was carried out for five participants (P_2_:P_6_) individually. For comparison, intra-session baseline SDG, using leave one out cross-validation (LOOCV) with target training data and target test data, was executed for each participant. The SFMG-DTL model obtained comparable estimation accuracies (R^2^ ≥ 88%) similar to the baseline SDG model (R^2^ ≤ 86%) across participants. Thus, performance with transfer learning obtained 2.4% improvement in estimating forces in dynamic SQ-2 motion. Moreover, the SFMG-DTL model encountered an error in estimation (NRMSE ≈ 0.6) that was 3.75% lower than the intra-session model across participants (mean MSE loss ≈ 5.14 N). Individual results of R^2^ and NRMSE (averaged for Model X and Model Y) are reported in Figure 8 for all five participants.

## 4. Discussions

### 4.1. Viability of Calibration

The pretrained SFMG-DTL model was further retrained with a few calibration data sets to adapt to the target domain. The model worked well for both SDA and SDG once fine-tuned with calibration/target training data. To investigate the effect of calibration during SDA, the pretrained model was evaluated on target test data without fine-tuning towards target distribution. It was interesting that the pretrained model without fine-tuning could predict forces in X dimension with higher estimation accuracy and lower error (R^2^ ≥ 89%, NRMSE ≈ 0.09%) although it could not estimate well in Y dimension (R^2^ ≤ 12%, NRMSE ≥ 8%) with no adaptation to target domain. For SDG, similar trends were observed in X dimension (R^2^ ≥ 89%, NRMSE ≈ 0.09%) and Y dimension (R^2^ ≤ 25%, NRMSE ≥ 6%). Muscle contractions in extension/flexion (X dimensions) and abduction/adduction (Y dimensions) could affect FSR readings and model’s performances although this would require further study. Therefore, it was revealed that fine-tuning with calibration data was mandatory for estimating forces in 2D planar SQ-1 motion for SDA as well as in SQ-2 motion for SDG.

For compliant collaboration, applied forces in both dimensions were needed to be estimated well simultaneously. Therefore, the proposed framework would not work without calibration data. The calibration data represented the instantaneous FMG data of muscle contraction during interactions, and it was found as an effective way to include the current state of muscle readings in certain activities during pHRI. Additionally, using fewer calibration data sets was helpful, as the model was calibrated within few minutes.

### 4.2. Viability of SDG

**Figure 9:**
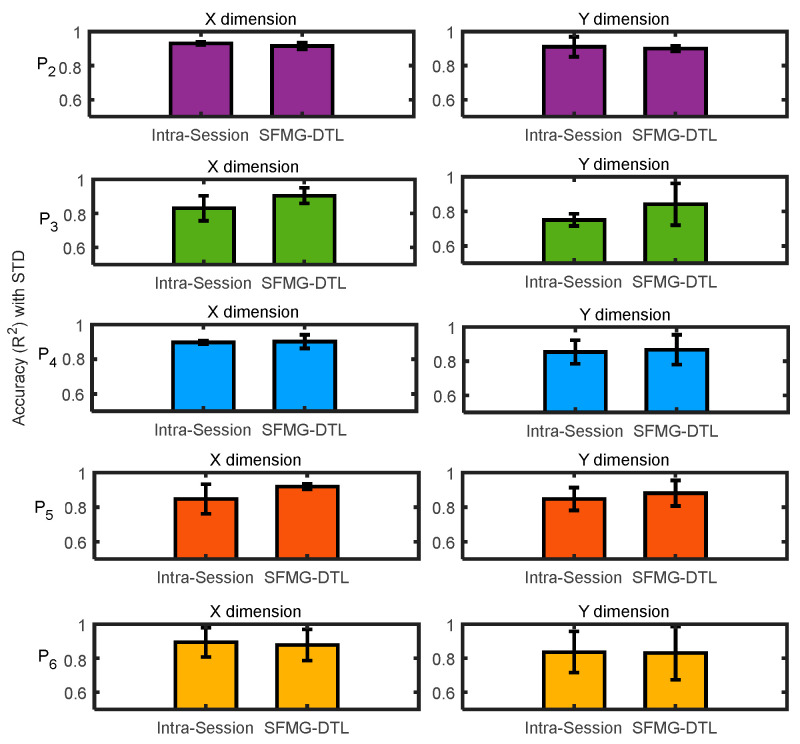
Comparing SFMG-DTL model with intra-session evaluation on case ii: target dataset 2 model in estimating forces in X and Y dimensions in domain generalization.

In this case, estimation accuracies and errors obtained by SFMG-DTL model were found comparable with intra-session evaluation of baseline SDG for participants P_2_ and P_6_, while it performed better for P_3_–P_5_. Although the overall performance improvement was limited, it was interesting that the SFMG-DTL model improved accuracies in estimating force in the Y dimension compared to the baseline SDG model for some participants, as shown in Figure 9. A *t*-test was carried out with a 95% confidence level to compare performances of the intra-session and the SFMG-DTL model. Estimation accuracies (R^2^) in Y-dimension via the SFMG-DTL model were found statistically significant. This would improve designing FMG-based HMI in future practical applications.

## 5. Conclusions

Estimating applied hand force using force myography (FMG) can be effective yet challenging due to the transient, time-variant nature of the bio signal. Controlling machines using data-driven models in HMI or pHRI over multiple days are affected by sensor position shifts and/or physiological effects. This study investigated multiple sessions of labelled FMG data to overcome such inherent challenges by pretraining a deep learning model using a CNN algorithm. Calibration data from individual participants allowed the pretrained model to be fine-tuned towards individual target distribution and to adapt the target task. The proposed SFMG-DTL model was evaluated in both domain adaptive and domain-generative transfer learning scenarios and obtained better prediction accuracies and lower losses. The model obtained estimation accuracies (R^2^) of 89% and 88.4% in SDA and SDG, respectively. In both cases, SFMG-DTL outperformed the SVR, MSVR, and intra-session models. Performance of the pretrained deep transfer model achieved improvements over the intra-session model for both intra-subject and cross-subject evaluations (6% and 2.4% increase in estimation in SDA and SDG, respectively).

Although SDA and SDG showed potential improvements, achieving these in real-time situations needs to be examined. In addition, in practical scenarios, collecting labelled data is not easy or sometimes impossible. Therefore, unsupervised domain adaptations in challenging situations could be investigated in the future studies. The SFMG-DTL model performed well for domain generalization but was limited to a certain pHRI collaborative task. A pretrained model using more diversified source domains would play a vital role in improving domain generalization and extend to all other possible interactions. Such a pretrained model can be useful for unseen target domains where the target label data are scarce or inadequate in real scenarios. Moreover, an FMG-based transfer learner can be more practical for domain adaptation to implement an FMG-based application either for one-time or periodic usage by overcoming sensor position shifts on multiple elapsed days.

## Figures and Tables

**Table 1:** Acronyms used.

Acronyms	Meaning	Acronyms	Meaning
SDA	Supervised domain adaptation	SQ-1	Interaction force in square motion with variable sizes in domain adaptation
SDG	Supervised domain generalization	SQ-2	Interaction force in square motion in domain generalization
Ds	Source domain	D_t-SDA_, T_t-SDA_	Target domain and target task in inter-session SDA
Dt	Target domain	D_t-SDG_, T_t-SDG_	Target domain amd target task in inter-participant SDG
Ts	Source task	D_si_	Multiple source domains
Tt	Target task	F_xt_^’^	Estimated applied forces in X dimension
C_d_	Calibration data	F_yt_^’^	Estimated applied forces in Y dimension

**Table 2:** Source and target domains.

Pretraining Phase		Evaluation Phase			
Source Domain	Hyper Parameters	Target Domain	Hyper Parameters	Fine Tuning	Target Test Data
$Ds_{i}$ = {X_s_, Y_s_} {P_1_}, where, $Ds_{i}=\left\{Ds_{1}\cup^{\;}Ds_{2}\cup^{\;}Ds_{3}\right\}$ = 8400 × 32 samples, T_SDA_: applied force in SQ-1 motion	SGD Epochs: 40 LR: 1E-4	case i. D_t-SDA_= {X_s_, Y_s_} {P_1_} where T_SDA_: applied force in SQ-1 motion	SGD Epochs: 60 LR: 1E-5	C_d_ = {X_c_, Y_c_} 1200 × 32 samples	D_t_ = {X_t_, Y_t_} 400 × 32 samples
		case ii. D_t-SDG_ = {X_s_, Y_s_} {P_2_, …, P_6_}, where T_SDG:_ applied force in SQ-2 motion			

## Data Availability

Data are available upon request to the corresponding author.
